# Characterization of ST14A Cells for Studying Modulation of Voltage-Gated Calcium Channels

**DOI:** 10.1371/journal.pone.0132469

**Published:** 2015-07-06

**Authors:** Mandy L. Roberts-Crowley, Ann R. Rittenhouse

**Affiliations:** Department of Physiology, Program in Neuroscience, University of Massachusetts Medical School, Worcester, Massachusetts, United States of America; Vanderbilt University Medical Center, UNITED STATES

## Abstract

In medium spiny neurons (MSNs) of the striatum, dopamine D_2_ receptors (D_2_Rs) specifically inhibit the Ca_v_1.3 subtype of L-type Ca^2+^ channels (LTCs). MSNs are heterogeneous in their expression of dopamine receptors making the study of D_2_R pathways difficult in primary neurons. Here, we employed the ST14A cell line, derived from embryonic striatum and characterized to have properties of MSNs, to study Ca_v_1.3 current and its modulation by neurotransmitters. Round, undifferentiated ST14A cells exhibited little to no endogenous Ca^2+^ current while differentiated ST14A cells expressed endogenous Ca^2+^ current. Transfection with LTC subunits produced functional Ca_v_1.3 current from round cells, providing a homogeneous model system compared to native MSNs for studying D_2_R pathways. However, neither endogenous nor recombinant Ca_v_1.3 current was modulated by the D_2_R agonist quinpirole. We confirmed D_2_R expression in ST14A cells and also detected D_1_Rs, D_4_Rs, D_5_Rs, G_q_, calcineurin and phospholipase A_2_ using RT-PCR and/or Western blot analysis. Phospholipase C β-1 (PLCβ-1) expression was not detected by Western blot analysis which may account for the lack of LTC modulation by D_2_Rs. These findings raise caution about the assumption that the presence of G-protein coupled receptors in cell lines indicates the presence of complete signaling cascades. However, exogenous arachidonic acid inhibited recombinant Ca_v_1.3 current indicating that channels expressed in ST14A cells are capable of modulation since they respond to a known signaling molecule downstream of D_2_Rs. Thus, ST14A cells provide a MSN-like cell line for studying channel modulation and signaling pathways that do not involve activation of PLCβ-1.

## Introduction

Two classes of L-type Ca^2+^ channel (LTC) α_1_ subunits are expressed in the brain: α_1C_ (Ca_V_1.2) and α_1D_ (Ca_V_1.3) [1] with highest expression in cerebral cortex and striatum [2]. While differing in biophysical properties and pharmacological sensitivities, both LTCs contribute to membrane excitability, synaptic regulation and gene transcription [3]. In turn, neurotransmitters act via G-protein coupled receptors (GPCRs) to modulate membrane excitability and alter transfer of information within neural circuits. Modulation of LTCs by dopamine GPCR signaling pathways is important in medium spiny neurons (MSN) of the striatum since these neurons are the only source of output from the striatum [4] and are adversely affected in both Parkinson’s and Huntington’s Diseases [5, 6].

Two families of dopamine receptors exist. The D_1_-like receptor family (D_1_R, D_5_R), couples to the G protein G_s_, enhancing L-current [7, 8] and the firing rate of MSNs [7]. Conversely, the D_2_-like receptor family (D_2_R, D_3_R, D_4_R) couples to G_i/o_ [9], inhibiting L-current [10] and the firing rate of MSNs [11]. Two heterogeneous groups of MSNs respond to dopaminergic input: D_1_R-expressing MSNs and D_2_R-expressing MSNs, which are associated with the direct and indirect output, respectively [6]. The balance of output pathways between the opposing D_1_R- and D_2_R-expressing MSNs coordinates motor control [12]. Consequently drugs developed to treat Parkinson’s disease target dopamine receptors, particularly D_2_Rs [13] and more recently LTCs [14, 15]. MSNs express both Ca_V_1.2 and Ca_V_1.3, but D_2_R activation inhibits only Ca_V_1.3 [11]. In Parkinson’s disease models, loss of D_2_R modulation of Ca_V_1.3 leads to loss of dendritic spines [16]. Therefore, the pathway underlying D_2_R modulation of LTC current appears critical for normal function; however due to dopamine receptor heterogeneity in MSNs, the molecular relationship between D_2_Rs and LTCs has been difficult to elucidate.

Moreover, two different mechanisms may mediate D_2_R inhibition of LTC current. One characterized pathway involves G_q_, phospholipase C (PLC), inositol triphosphate (IP_3_)-induced Ca^2+^ release, and protein phosphatase 2B (PP2B) also known as calcineurin [10]. Additionally, D_2_R activation releases arachidonic acid (AA) in vivo [17–20], in primary neurons [21] and in transfected cell lines [22]. Our laboratory has demonstrated that exogenously applied AA inhibits LTC currents in superior cervical ganglion neurons (SCG) [23–25]. These currents are most likely exclusively due to Ca_V_1.3 current [26]. Additionally, we have shown that AA inhibits recombinant Ca_V_1.3 currents when expressed in HEK293 cells [27]. Therefore, a second D_2_R signaling pathway inhibiting Ca_V_1.3 may involve activation of Ca^2+^-dependent cytosolic phospholipase A_2_ (cPLA_2_), which cleaves AA from phospholipids, similar to M_1_ muscarinic receptor (M_1_R) modulation of LTC current in SCG [25].

In the present study, we developed a model system to probe the D_2_R signaling pathway inhibiting Ca_V_1.3 using the ST14A cell line, created from embryonic rat striatum [28]. Retroviral transduction of the temperature-sensitive SV40 large T antigen enables ST14A cells to grow and divide at the permissive temperature of 33°C. At higher temperatures the cells differentiate to exhibit general neuronal, as well as specific MSN-like, properties including functional D_2_-like receptors [28, 29]. We examined whether ST14A cells express identified signaling molecules downstream of D_2_Rs in an effort to develop a model cell line with MSN properties to study the functional effects of D_2_R signaling on Ca_V_1.3. We found that round ST14A cells lacked endogenous Ca^2+^ current and exploited this deficiency by transfecting cells with Ca_V_1.3 and accessory subunits, thus eliminating the need for pharmacological blockers. Recombinant Ca_V_1.3 currents were characterized and tested for current modulation by the D_2_ dopamine agonist quinpirole and by exogenous AA.

## Materials and Methods

### Preparation of Cells

The ST14A cell line, created by Elena Cattaneo and described in Cattaneo and Conti, 1998 [28], was given to us as a gift from Dr. Michelle E. Ehrlich (Jefferson Medical School) with permission from Dr. Elena Cattaneo (University of Milano). ST14A cells were propagated at the permissive temperature of 33°C in Dulbecco’s modified Eagle’s medium (DMEM; Gibco) supplemented with 0.11 g/L sodium bicarbonate (Sigma, St. Louis, MO), 0.29 g/L L-glutamine, 3.9 g/liter HEPES, 100 units/ml penicillin-streptomycin, and 10% fetal bovine serum (FBS; Invitrogen, Carlsbac, CA). Cells were transferred to 37°C to promote differentiation and used 1–2 days later. The A9L cell line, derived from A9 L cells co-transfected with the human D_2_R and obtained from the American Type Culture Collection (ATCC, Manassas, VA), were propagated at 37°C in DMEM with 4 mM L-glutamine adjusted to contain 1.5 g/L sodium bicarbonate and 4.5 g/L glucose containing 10% FBS. HEK 293 cells were propagated at 37°C in DMEM/F12 containing 10% FBS. All cells were maintained in a temperature-controlled humidified incubator at 5% CO_2_ and passaged once flasks became 80–90% confluent. SCG, striatum and cortex of 1 to 4-day old or adult Sprague-Dawley rats (Charles River Laboratories, Wilmington, MA) were isolated following CO_2_ exposure and decapitation using a protocol (protocol # 822) approved by the Institutional Animal Care and Use Committee (IACUC) of University of Massachusetts Medical School. This study was carried out in strict accordance with the recommendations in the Guide for the Care and Use of Laboratory Animals of the National Institutes of Health. All efforts were made to minimize animal suffering. The Institutional Animal Care and Use Committee (IACUC) of University of Massachusetts Medical School specifically approved animal use for this study.

### Electrophysiology

Whole-cell currents were recorded at room temperature (RT, 20–24°C) with an Axon 200B patch clamp amplifier (Molecular Devices, Sunnyvale, CA). Currents were filtered at 1–5 kHz and digitized at 5 times the filter cut-off frequency of the 4-pole Bessel filter of the amplifier. Electrodes were pulled from borosilicate glass capillary tubes and each electrode was fire-polished to ~1 m to give the pipette a resistance of 2–3 M. The pipette solution consisted of (in mM): 125 Cs-Aspartate, 10 HEPES, 0.1 BAPTA, 5 MgCl_2_, 4 ATP and 0.4 GTP brought to pH 7.50 with CsOH. High resistance seals were established in Ca^2+^ Tyrode’s consisting of (in mM): 5 CaCl_2_, 145 NaCl, 5.4 KCl, and 10 HEPES brought to pH 7.50 with NaOH. Once a seal was established and the membrane ruptured, the Tyrode’s solution was exchanged for an external bath solution consisting of (in mM): 125 NMG-Aspartate, 20 Ba^2+^, 10 HEPES brought to pH 7.50 with CsOH.

FPL 64176 and nimodipine were prepared as stock solutions in 100% ethanol and stored at -20°C. AA (5,8,11,14-eicosatetraenoic acid; NuCheck Prep, Elysian, MN) and oleic acid (NuCheck Prep) were dissolved in 100% ethanol and stored under nitrogen as stock solutions at -70°C. ω-conotoxin GVIA (Bachem, Torrence, CA) and quinpirole were prepared as stock solutions in double distilled water and stored at -70°C. Oxotremorine-M (Tocris, Ellisville, MO) was prepared fresh daily by making a 10 mM stock in double distilled water. Working dilutions were made fresh daily by diluting stock solutions at least 1:1,000 with external bath solution. For ethanol prepared stocks, the final ethanol concentration was less than 0.1%. Bovine serum albumin (BSA; fraction V, heat shock, fatty acid ultra-free; Roche Applied Science, Indianapolis, IN) was added directly to the bath solution for a final concentration of 1 mg/ml. All chemicals were purchased from Sigma unless otherwise noted.

Data were acquired using Signal 2.14 software (Cambridge Electronic Design, Cambridge, England) and stored for later analysis on a personal computer. Linear leak and capacitive currents were subtracted from all traces. Data are presented as the mean ± s.e.m. Significance was determined statistically using a two-tailed paired or unpaired *t*-test, or a one-way ANOVA. Analysis programs include Signal (Cambridge Electronic Design), Excel (Microsoft, Redmond, WA) and Origin (OriginLab, Northampton, MA).

### Transfection

ST14A cells were transfected by lipofectamine (Invitrogen, Carlsbad, CA) with a 1:1:1 molar ratio of Ca_V_1.3, β_2a_, and α_2_δ subunits [30]. Constructs for Ca_V_1.3b (+exon11, Δexon32, +exon42a; GenBank accession #AF370009), and α_2_δ-1 (GenBank accession #AF286488) were a gift from Dr. Diane Lipscombe (Brown University) and the construct for Ca_V_β_2a_ (GenBank accession #M80545) was a gift from Dr. Edward Perez-Reyes (University of Virginia). For all transfections, 0.4 μg of DNA was used per well of a 12-well plate. Prior to transfection, cells were washed with DMEM. The DNA mixture was then added dropwise to each well, gently swirled then incubated for 1–3 h at 37°C in a 5% CO_2_ incubator. Supplemented media, without antibiotics, was returned to the wells to bring the volume up to 1 ml (normal growth medium volume). Cells were washed with full media 2 and 4 h later and assayed for transient gene expression after 24–72 h.

### Reverse Transcriptase-Polymerase Chain Reaction

Homogenized tissue samples (50–100 mg) or confluent cells in a 100 mm dish were lysed in 1 ml TRIzol Reagent (Invitrogen). RNA was separated from DNA by phenol-chloroform phase separation. RNA was precipitated with isopropyl alcohol and washed with 75% ethanol. The RNA pellet was dried and resuspended in RNase-free water. RNA samples had an A_260_/A_280_ ratio between 1.6 and 1.8 and were treated with DNase to eliminate contamination with genomic DNA. For reverse transcription, cDNA was synthesized from the mRNA by adding 1 μl 10X buffer RT, 1 μl dNTP Mix (5 mM each dNTP), 1 μl Oligo-dT primer (0.5 mg/ml, Promega, Madison, WI), 0.125 μl RNase Inhibitor (40 U/μl, Promega), 0.5 μl Omniscript Reverse Transcriptase (4 U/μl) and RNase-free water for a total volume of 10 μl (all reagents from QIAGEN, Valencia, CA, unless otherwise noted). The mixture was incubated at 37°C for 1 h. The mixture was then heated at 93°C for 5 min and then placed on ice to inactivate the transcriptase. PCR amplification was then performed with a Techgene thermal cycler (Techne Inc, Burlington, NJ) with thin walled PCR tubes.

PCR primers for dopamine receptors D_1_-D_5_ (D_5_ formerly referred to as D_1b_) were sequences previously published [31]. D_1_: 5’-GAC AAC TGT GAC ACA AGG TTG AGC-3’ and 5’-ATT ACA GTC CTT GGA GAT GGA GAT GGA GCC-3’ yields a 609 base pair (bp) product. D_2_: 5’-GCA GTC GAG CTT TCA GAG CC-3’ and 5’-TCT GCG GCT CAT CGT CTT AAG-3’ yields 404 and 317 bp products recognizing the long and short forms of the D_2_R, respectively. D_3_: 5’-AGC ATC TGC TCC ATC TCC AAC CC-3’ and 5’-A GGA GTT CCG AGT CCT TTC CAC G-3’ yields a 461 bp product. D_4_: 5’-TC ATG CTA CTG CTT TAC TGG GCC A-3’ and 5’-T CTG AGA GAG GTC TGA CTC TGG TC-3’ yields a 223 bp product. D_5_: 5’-AGT CGT GGA GCC TAT GAA CCT GAC-3’ and 5’-GCG TCG TTG GAG AGA TTT GAG ACA-3’ yields a 517 bp product. GAD65 and GAD67 primers were from sequences previously published [32]. GAD65: 5’-CGC CCC TGT ATT TGT ACT AC-3’ and 5’-GCC AAG AGA GGA TCA AAA GC-3’ yields a 400 bp product. GAD67: 5’CAC ACC AGT TGC TGG AAG-3’ and 5’- ACA AAC ACG GGT GCA ATT-3’ yields 318 and 238 bp products. GAPDH primers were from sequences previously published [33]. GAPDH: 5’-TGC CAA GGC TGT GGG CAA GG-3’ and 5’-GCT TCA CCA CCT TCT TGA TG-3’ yields a 199 bp product and confirmed equal loading.

Reaction mixtures for PCR contained 200 ng of cDNA, 5 μl of 2.5 X Eppendorf MasterMix [Taq DNA Polymerase (62.5 U/ml), 125 mM KCl, 75 mM Tris-HCl, 3.75 mM Mg(OAc)_2_, 0.25% Igepal-CA630, 500 μM of each dNTP and stabilizers, Brinkmann, Westbury, NY], 1 μl each of forward and reverse primer (5 pmol/ μl, Invitrogen for dopamine receptors and GAD; Qiagen for GAPDH), 2 to 4 mM Mg^2+^ (Brinkmann) and distilled water to a final volume of 12.5 μl. High (4 mM) Mg^2+^ concentrations were necessary to amplify dopamine receptor mRNAs. The protocol for dopamine receptor amplification was 94°C for 1 min, 58°C for 1 min, and 72°C for 3 min for 20 cycles [31]. For the D_3_R, an annealing temperature of 62°C was also tried. For GAD amplification, the protocol was 94°C for 3 min, 53°C for 1 min, 52°C for 1 min, 51°C for 1 min, and 70°C for 2 min for 30 cycles [32]. A 2 μl aliquot was used as a template for a second round of amplification for thirty cycles. PCR products and a 100 bp DNA ladder (Invitrogen; bright band 600 bp or Promega, Madison, WI; bright band 500 bp) were separated by electrophoresis in 2% agarose gels stained with ethidium bromide. Bands excised from gels were sequenced by the UMASS Medical School Nucleic Acid Facility and compared to published sequences (GenBank accession number): D_1_ (M35077), D_2_ (M36831), D_4_ (M84009), D_5_ (M69118), GAD65 (M74826), and GAD67 (M81883).

### Western Blot Analysis

The immunoblot protocol has been previously described [34]. All extracts and buffers contained protease inhibitors (Santa Cruz Biotechnology, Santa Cruz, CA): pepstatin A (1μg/ml), leupeptin (10 μg/ml), aprotinin (20 μg/ml), phenylmethanesulfonyl fluoride (200 nM), and calpain inhibitor I and II (8 μg/ml each). Day 4 rat brain or cells grown in uncoated 60-mm dishes were solubilized in lysis buffer (0.15 M NaCl, 5 mM EDTA, 1% Triton-X 100, 10 mM Tris-Cl pH 7.4), sonicated, and centrifuged to remove insoluble material. Protein concentration was determined by the RC/DC assay (Bio-Rad, Hercules, CA). 20–30 μg of protein was loaded per lane and separated on 8% SDS-polyacrylamide gels. Equal protein loading was confirmed by staining with β-actin antibodies.

Antibodies used include: *monoclonal*- D_2_R (1:400; Santa Cruz Biotechnology), PP2B and β-actin (1:500 and 1:10,000; Sigma); *polyclonal*- M_1_R and G_q_α (1:1,000; Santa Cruz Biotechnology), cPLA_2_ (1:200; Cell Signaling Technology, Danvers, MA), PLCβ-1 (1:200; Millipore, Billerica, MA); *secondary*- HRP-conjugated goat anti-mouse or bovine anti-rabbit (1:15,000 or 1:10,000; Santa Cruz Biotechnology). Primary and secondary antibodies were diluted with PBS.

## Results

**Fig 1:**
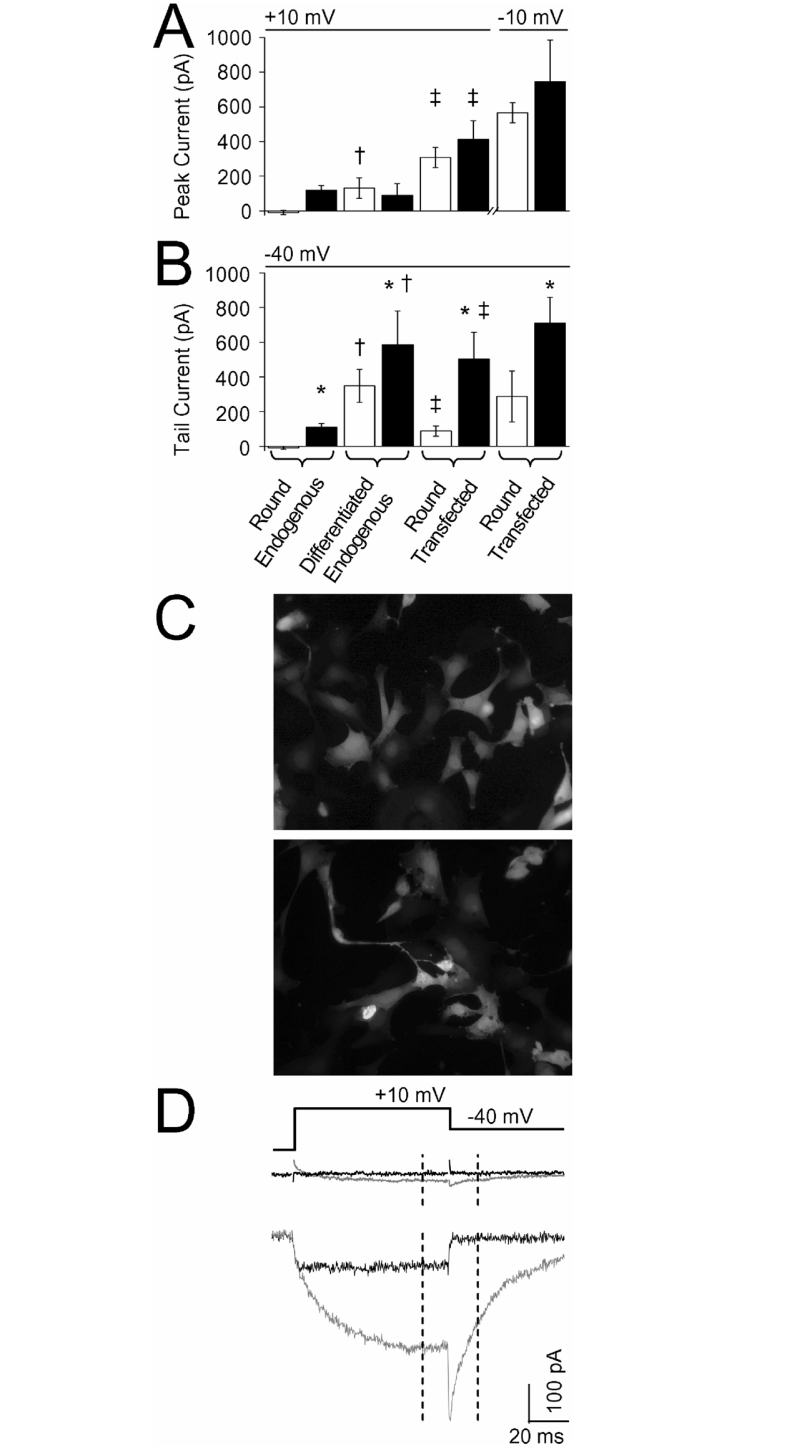
ST14A cells have endogenous Ca^2+^ currents but are capable of expressing recombinant current. (A-B) Currents were recorded from round (undifferentiated, no processes) or differentiated (having processes) endogenous ST14A cells. Note: round cells from ST14A cells grown at either 33°C or 37°C exhibited little to no current and were pooled. Currents were recorded from round ST14A cells grown at 33°C, transfected and grown at 37°C for at least 24 hours before recording. Each set of cells were recorded in the absence (white bars) and presence of 1 μM FPL (black bars). Summary of peak Ca^2+^ currents measured from a holding potential of -90 mV to a test potential of +10 mV (A) and then to a tail potential of -40 mV (B). Endogenous current from differentiated cells was significantly larger than endogenous current from round cells (†; *p* < 0.05); Transfected current was significantly larger than endogenous current from round cells (‡; *p* < 0.05); FPL significantly increased differentiated endogenous and transfected tail current (*; *p* < 0.05), n = 7–15. (C) Transfected ST14A cells expressed GFP throughout the cell soma and in a small percentage of differentiated cells, in the processes. Images (20X magnification) were captured ~24 hours post-transfection. The transfection rate for these cells was ~50%. (D) *Top*: Protocol for eliciting currents from a holding potential of -90 mV to the test potential of +10 mV for 100 ms before repolarizing to -40 ms. Individual traces from round endogenous (*middle*) or round transfected (*bottom*) cells. Dashed lines indicate where peak and tail current were measured 65 ms after depolarization and 15 ms after repolarization, respectively. In the presence of LTC agonist, 1 μM FPL, both the peak and long-lasting tail current increase (gray trace) and display slowed activation and deactivation kinetics, characteristic of FPL-induced L-current.

To determine if ST14A cells express endogenous Ca^2+^ current, we measured whole-cell currents from cells grown at 33 or 37°C. Two populations of cells were distinguished based on morphology: round or differentiated, with the definition of having neuron-like projections. From a holding potential of -90 mV, round cells exhibited zero to little endogenous peak or tail current measured at +10 mV or -40 mV respectively, regardless of the temperature at which the cells were grown (Fig 1A). Differentiated cells had significantly more endogenous current than round cells at both potentials (Fig 1B). Application of the LTC agonist, FPL 64176 (FPL; 1 μM) enhanced endogenous currents at both potentials (Fig 1A and 1B), indicating that at least a component of the endogenous current was LTC. Since round cells showed little to no LTC current, we transfected the ST14A cells with LTC channel subunits, Ca_V_1.3, β_2a_, and α_2_δ-1, along with green fluorescent protein (GFP) and recorded whole-cell currents from green fluorescing round cells (Fig 1C). Peak and tail currents from round transfected cells were significantly larger than those recorded from round untransfected cells (Fig 1A and 1B). FPL significantly enhanced recombinant current at -40 mV. Fig 1D shows representative individual traces of round endogenous or round recombinant current before and after application of FPL. The recombinant current shows little to no inactivation with 20 mM Ba^2+^ as the charge carrier [35], typical of LTC current coexpressed with the accessory subunit, β_2a_ [36].

Since Ca_V_1.3 activates at more negative voltages compared to Ca_V_1.2 [30], we measured peak current at -10 mV. Recombinant current recorded at -10 mV was larger than at +10 mV (Fig 1A) and this increase was also reflected in the tail current amplitude at -40 mV (Fig 1B). The summary of these results shows that round ST14A cells have little endogenous LTC current but are capable of being transfected with LTCs and expressing functional LTC currents. Therefore, the round cells represent a population of ST14A cells that we used to study recombinant LTC function in isolation from other types of native Ca^2+^ channels.

**Fig 2:**
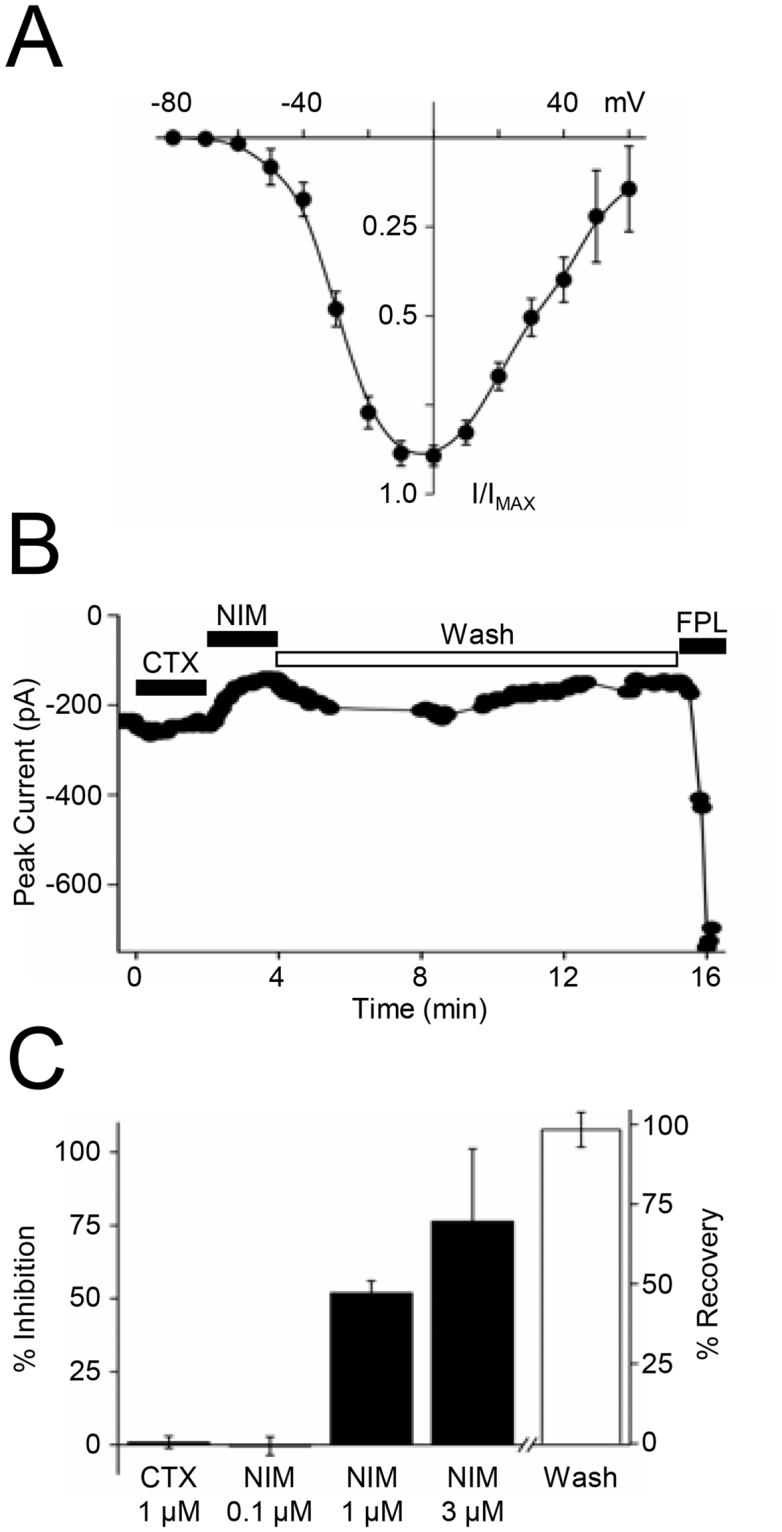
Whole-cell currents from transfected ST14A cells have biophysical and pharmacological properties of Ca_V_1.3 LTCs. (A) The current-voltage (I-V) relationship shows that recombinant Ca_V_1.3 current activates at approximately -60 mV; n = 15. (B) Example time course of peak current at -10 mV. 1 μM ω-conotoxin GVIA (CTX, an N-type Ca^2+^ channel antagonist) was added for 2 min. Bath solution was exchanged with 1 μM nimodipine (NIM, an LTC antagonist) for 2 min or until a new stable baseline was reached and then washed off to show reversibility. FPL (1 μM) was added at the end of the recording. (C) Summary of pharmacological inhibition of transfected current; n = 3–6.

We characterized recombinant Ca_V_1.3 current further to determine whether transfected channel activity in ST14A cells exhibits biophysical and pharmacological properties of Ca_V_1.3 observed in oocytes and HEK 293 cells [30]. First, we measured peak current across a range of voltages to show that channels open at relatively negative voltages compared to Ca_V_1.2 [30]. Indeed, in 20 mM Ba^2+^, Ca_V_1.3 currents activated at a test potential of -60 mV and peaked at -10 mV to 0 mV (Fig 2A). Recombinant current in ST14A cells exhibited a Ca_V_1.3 LTC pharmacological profile. Fig 2B shows a time course of the effects of Ca^2+^ channel ligands on Ca_V_1.3 current. The current was insensitive to the N-type Ca^2+^ channel antagonist, ω-conotoxin GVIA (1 μM; CTX) but was inhibited in a concentration-dependent manner by the LTC antagonist, nimodipine (0.1–3.0 μM; NIM). The inhibition produced by 1.0 μM NIM (51.8 ± 4%) is characteristic of the low-voltage sensitivity of Ca_V_1.3 compared to Ca_V_1.2, which would be fully blocked by 1.0 μM NIM [30]. Inhibition fully reversed by washing with bath solution. After wash out, channels remained sensitive to FPL. The antagonist data are summarized in the bar graph in Fig 2C. These findings show that recombinant current in ST14A cells displayed both current-voltage and pharmacological profiles specific to Ca_V_1.3 channels.

**Fig 3:**
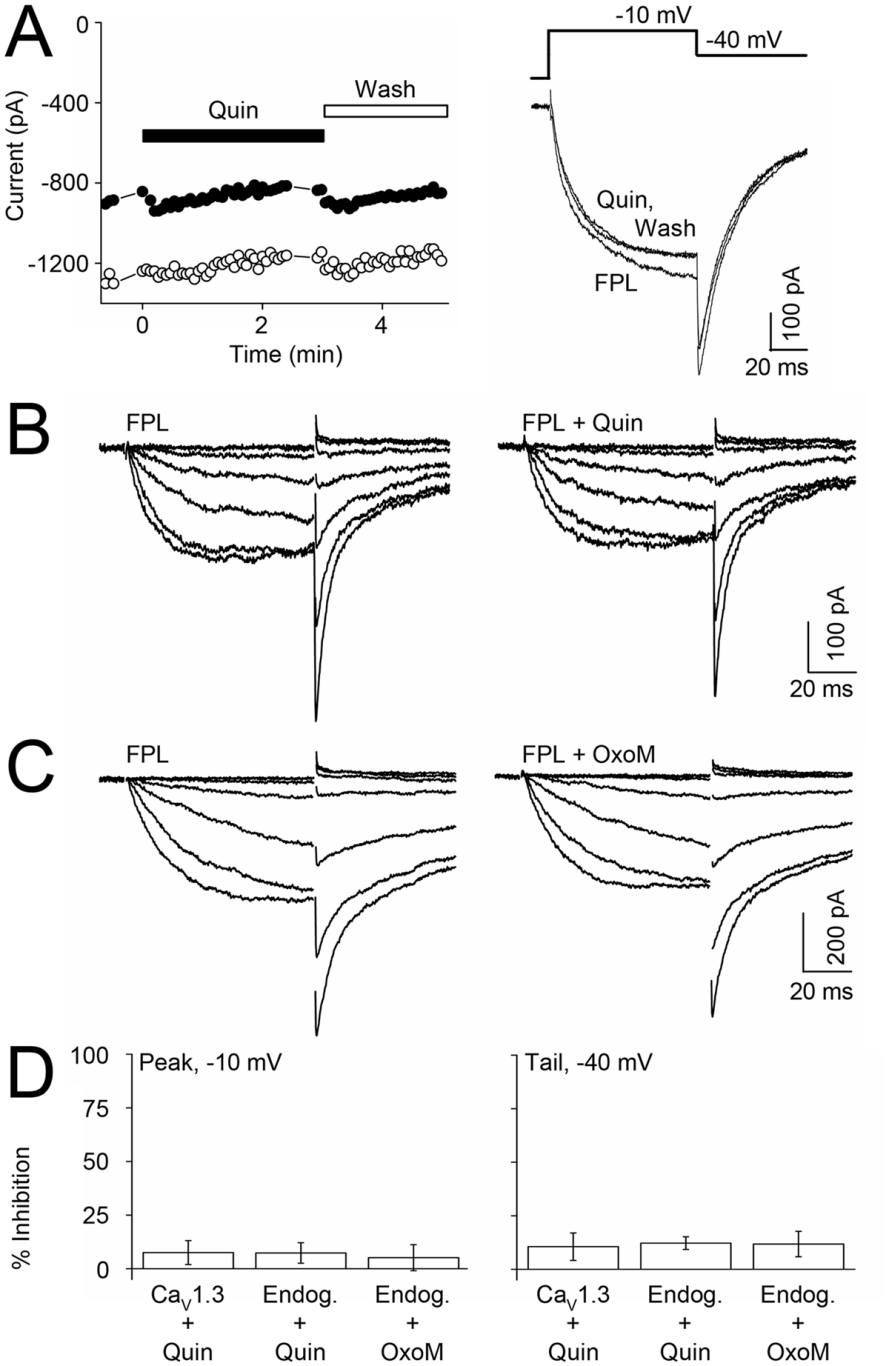
ST14A endogenous or recombinant Ca_V_1.3 current is not modulated by GPCR activation. (A) Time course (*left*) and representative sweeps (*right*) of recombinant Ca_V_1.3 current before (FPL), 1 min following 10 μM quinpirole application (Quin) and after removing Quin (Wash). (B–C) Endogenous, differentiated current traces in the presence of FPL across a range of test potentials (-50 mV to 0 mV) before (*left*) and after (*right*) application of (B) 10 μM Quin or (C) 10 μM oxotremorine M (Oxo-M). (D) Summary of percent inhibition of peak (*left*) and long-lasting tail (*right*) recombinant Ca_V_1.3 or endogenous currents by D_2_R or M_1_R agonist; n = 3–7.

To determine whether D_2_R activation by the agonist quinpirole (Quin) inhibits Ca_V_1.3 currents in ST14A cells, we recorded recombinant currents in the presence of FPL to enhance current amplitude. At a concentration of 10 μM, Quin had no significant effect on peak or tail current amplitude over time, (Fig 3A *left*) or in individual traces (Fig 3A *right*). To determine if only recombinant Ca_V_1.3 current was insensitive to D_2_R activation, we tested whether endogenous ST14A current from differentiated cells could undergo modulation. Again 10 μM Quin had no significant effect on endogenous peak or long-lasting tail current amplitude. Fig 3B shows representative current traces in the presence of 1 μM FPL from a range of voltages before and after Quin. Since a majority of MSNs express muscarinic M_1_ receptors (M_1_Rs) as well as dopamine receptors [37], we tested whether activation of this receptor would inhibit endogenous current. The muscarinic agonist oxotremorine-M (Oxo-M; 10 μM) had no effect on endogenous peak or long-lasting tail current amplitude. Fig 3C shows representative current traces in the presence of FPL from a range of voltages (-60 mV to -10 mV) tested before and after Oxo-M. Fig 3D summarizes the effect of Quin and Oxo-M on peak (*left*) and long-lasting tail current (*right*) from recombinant Ca_V_1.3 versus endogenous ST14A current. These results suggest that the D_2_R and M_1_R signaling pathways, which inhibit LTC current in MSNs, are not intact in ST14A cells. However, application of dopamine or Quin, increases CREB phosphorylation in ST14A cells, indicating that D_2_Rs do couple to intact signaling cascades such as the adenylyl cyclase and MAPK pathways [29].

**Fig 4:**
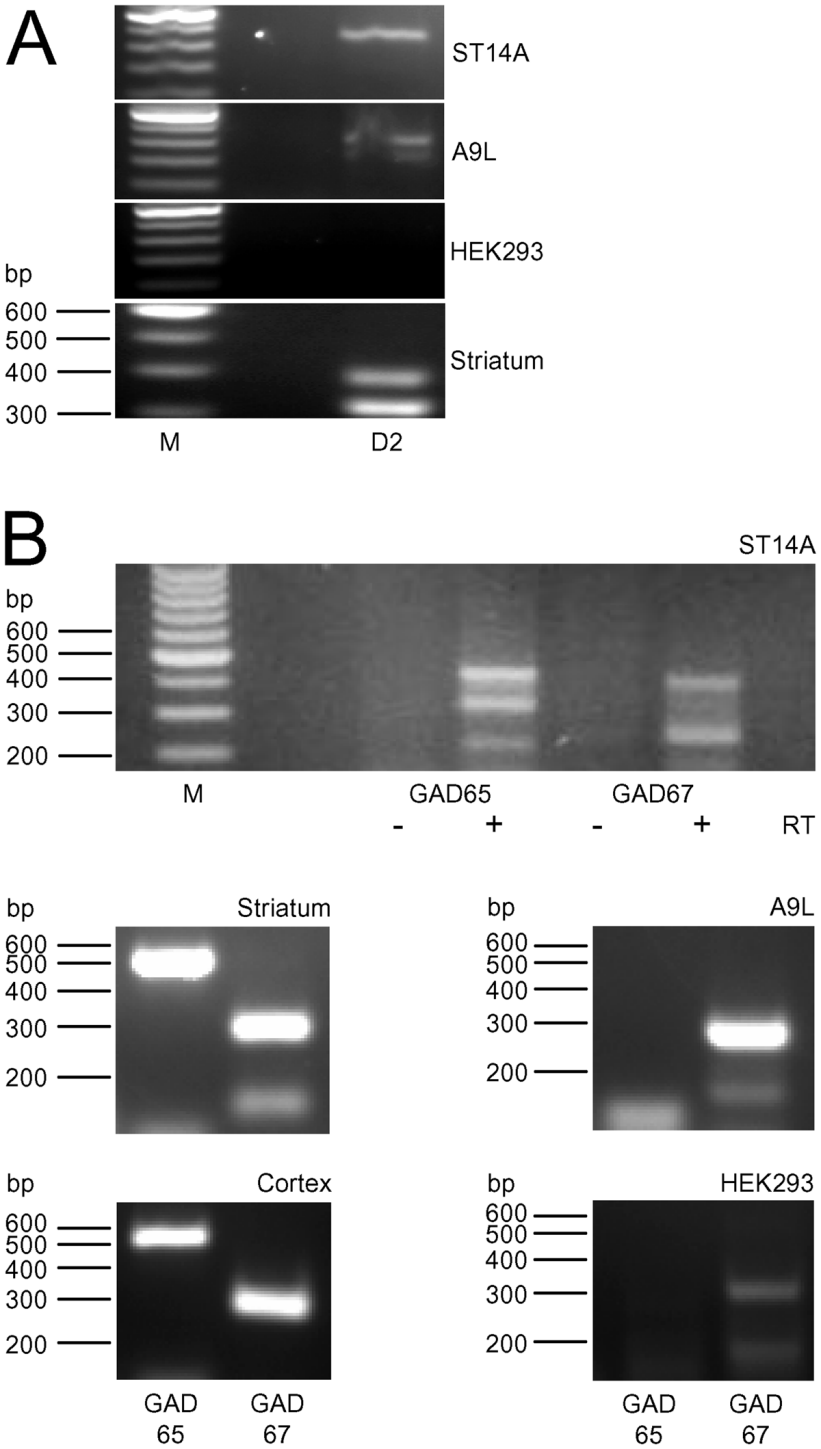
ST14A cells express several D_2_R family mRNAs. ST14A mRNA transcripts were amplified by RT-PCR. (A) D_2_R mRNA expression in ST14A, A9L, HEK 293 cells and in striatum. A9L cells that express D_2_Rs and striatum served as positive controls. Left lane contains a 100 bp ladder; brightest band is 600 bp. The band for ST14A cells was sequenced and BLAST search results matched previously published sequences for D_2_Rs. No D_2_R bands were detected in HEK 293 cells. (B) GAD65/67 mRNA expression in ST14A, striatum, cortex, A9L and HEK 293 cells. As a control, ST14A mRNA was not reverse-transcribed (- RT).

A non-selective D_2_R-like agonist, Quin also activates D_3_ and D_4_Rs [38]. Subsequently, D_3_ and D_4_Rs modulate immediate early gene expression [39, 40] raising the question of the identity of dopamine receptors in ST14A cells. Using RT-PCR, we examined dopamine receptor (D_1_, D_2_, D_3_, D_4_, and D_5_) mRNA content in ST14A cells, striatum (positive control for all dopamine receptors), cortex, A9L cells (D_2_R positive control; see Materials and Methods) or HEK 293 cells (negative control for all dopamine receptors). PCR products were detected for long and short splice variants of the D_2_R in ST14A cells (n = 4/11 and 2/11, respectively, striatum (n = 1/2 and 2/2, respectively), cortex (n = 1/2 for both) and A9L cells (n = 2/5 and 2/5, respectively) as shown in Fig 4A. Additionally, D_1_R (n = 2/10), D_4_R (7/10), D_5_R (4/10) (data not shown) mRNAs were also detected in ST14A cells, regardless of the temperature at which cells were grown. D_1_R, D_4_R and D_5_R mRNAs were detected in striatum whereas D_1_R and D_4_R mRNAs were detected in the cortex (data not shown). D_4_R mRNA was also detected in A9L cells (n = 3/4). Experiments for D_3_R mRNA expression in striatum, cortex, and ST14A cell samples resulted in a smear despite several attempts to adjust the protocol. HEK 293 cells showed no expression of any of the dopamine receptors tested. These results show that ST14A cells express mRNA for more than one D_2_-like receptor; this finding could account for the previously reported D_2_R-like changes in pCREB [29].

Since MSNs are GABAergic, a defining characteristeric of ST14A cells being MSN-like would be expression of glutamic acid decarboxylase (GAD), the enzyme that catalyzes GABA synthesis from glutamate. Using RT-PCR, we measured whether ST14A cells express GAD2 and GAD1, two genes which encode GAD with molecular weights of 65 and 67 kDa, respectively. The primers used against GAD1 detect both embryonic and adult splice forms of GAD67. All three bands corresponding to GAD65 and embryonic and adult GAD67 were detected in ST14A cells (Fig 4B). Alternate lanes in which RNA samples were not reverse transcribed served as controls for genomic DNA contamination and yielded no product. Using striatal and cortical tissues as positive controls for the GAD genes, and in A9L cells, we detected all three forms of GAD as well. In contrast, no bands for GAD65 and only faint bands for GAD67 were detected in HEK 293 cells. The presence of GAD in ST14A cells further confirms that this cell line exhibits GABAergic characteristics of MSNs.

**Fig 5:**
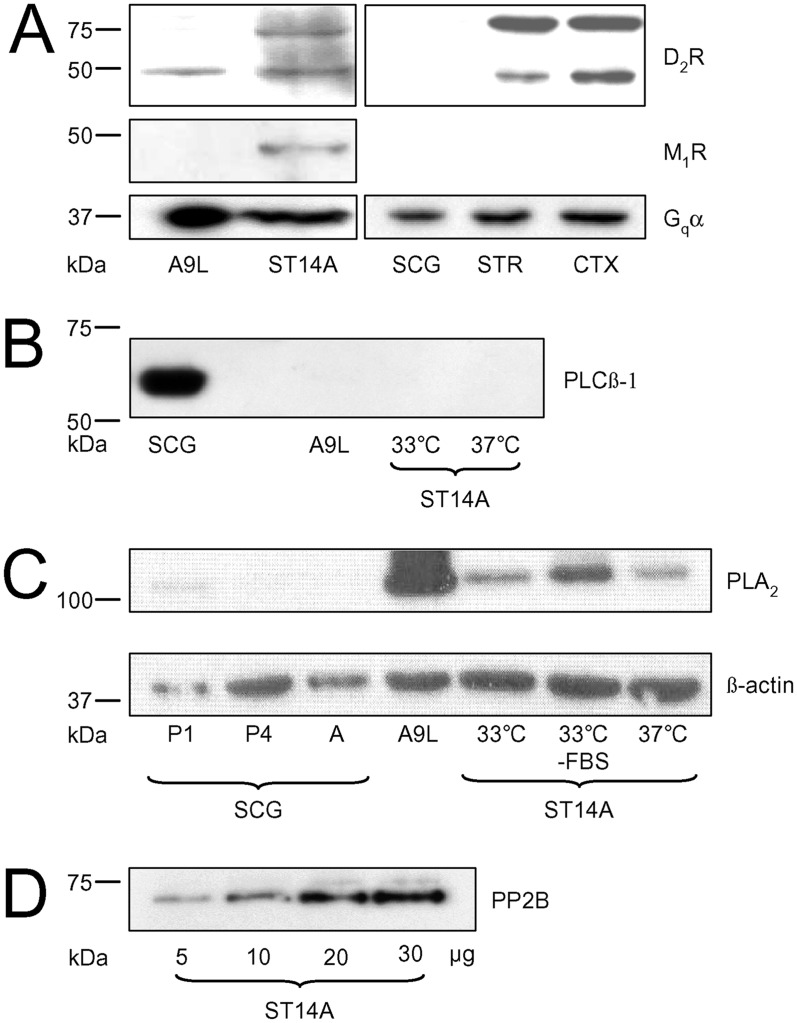
Expression of D_2_R and M_1_R signaling pathway proteins in ST14A cells. (A) *Top*: D_2_R expression in ST14A cells, A9L cells (positive control) and tissues: SCG (superior cervical ganglia), STR (striatum, positive control), CTX (cortex). The predicted molecular weight (MW) for the D_2_R is 50 kDa. The 75 kDa form may represent a glycosylated form of the D_2_R versus long and short D_2_R splice variants, which differ by ~40 amino acids and would not account for this MW difference. *Middle*: M_1_R expression in ST14A or A9L cells. The predicted MW of M_1_Rs is 50 kDa. *Bottom*: G_q_α expression in both cell lines and brain tissues. The predicted MW for G_q_α is 42 kDa. (B) PLCβ-1 expression, or absence, in SCG, A9L or ST14A cells at various temperatures. The antibody recognizes PLCβ-1 fragments at 100 and 41 kDa. (M, lane for MW ladder) (C) The presence of cPLA_2_ in SCG neurons at postnatal day 1 (P1) or 4 (P4) or adult (A), A9L and ST14A cells under varying conditions. The predicted MW for cPLA_2_ is 110 kDa. β-actin, MW of 42 kDa, is shown as a loading control. (D) PP2B in ST14A cells loaded at varying concentrations; predicted MW of the α-subunit is 61 kDa.

After confirming ST14A cells exhibit a similar mRNA expression profile for dopamine receptors and GAD as MSNs, we examined whether D_2_Rs and downstream signaling molecules were expressed in ST14A cells. Using Western blot analysis, D_2_R and M_1_R expression was confirmed in ST14A cells. D_2_R antibodies recognized a band at 50 kDa, the expected molecular weight of D_2_Rs (Fig 5A, *top panel*) for striatum and cortex (positive controls). A second band at 75 kDa, absent in the A9L cell line, most likely represents a glycosylated form of the receptor [41], further supporting the idea that the ST14A cells are similar to neurons in these brain regions regarding post-translational modification of proteins. Fig 5A (*middle panel*) shows that ST14A cells, but not A9L cells, express M_1_Rs, displayed as a 50 kDa band on Western blots. This antibody recognized M_1_R expression in SCG, striatum, and cortex [34]. Expression of G_q_α (Fig 5A, *bottom panel*), which couples to M_1_Rs, was also detected in the cell lines and tissue samples at the predicted molecular weight of 42 kDa. These results show that ST14A cells express D_2_R, M_1_R and G_q_α proteins.

Since D_2_Rs and M_1_Rs both couple to PLCβ-1, we examined whether ST14A cells express PLCβ-1. Protein expression of PLCβ-1 was not detected in either the A9L or ST14A cell lines, but was detected in SCG tissue (Fig 5B). The unanticipated absence of PLCβ-1 may account for the lack of Ca_V_1.3 modulation since PLCβ-1 is required for LTC current inhibition by both the D_2_R and M_1_R pathways [10, 42]. To determine the presence of molecules downstream of PLCβ-1 reported to participate in LTC modulation by these receptors [10, 25], we tested for cPLA_2_ and PP2B expression. Fig 5C shows that ST14A cells cultured at 33 and 37°C, as well as A9L cells, express cPLA_2_. cPLA_2_ was detected at low expression levels from postnatal day 1 SCG (positive control) as has been reported previously [25]. Fig 5D shows that ST14A cells also express PP2B. Since PLCβ-1 is downstream of both D_2_ and M_1_Rs, this result may explain our lack of inhibition of Ca_V_1.3 or endogenous LTC current by Quin or Oxo-M in ST14A cells.

**Fig 6:**
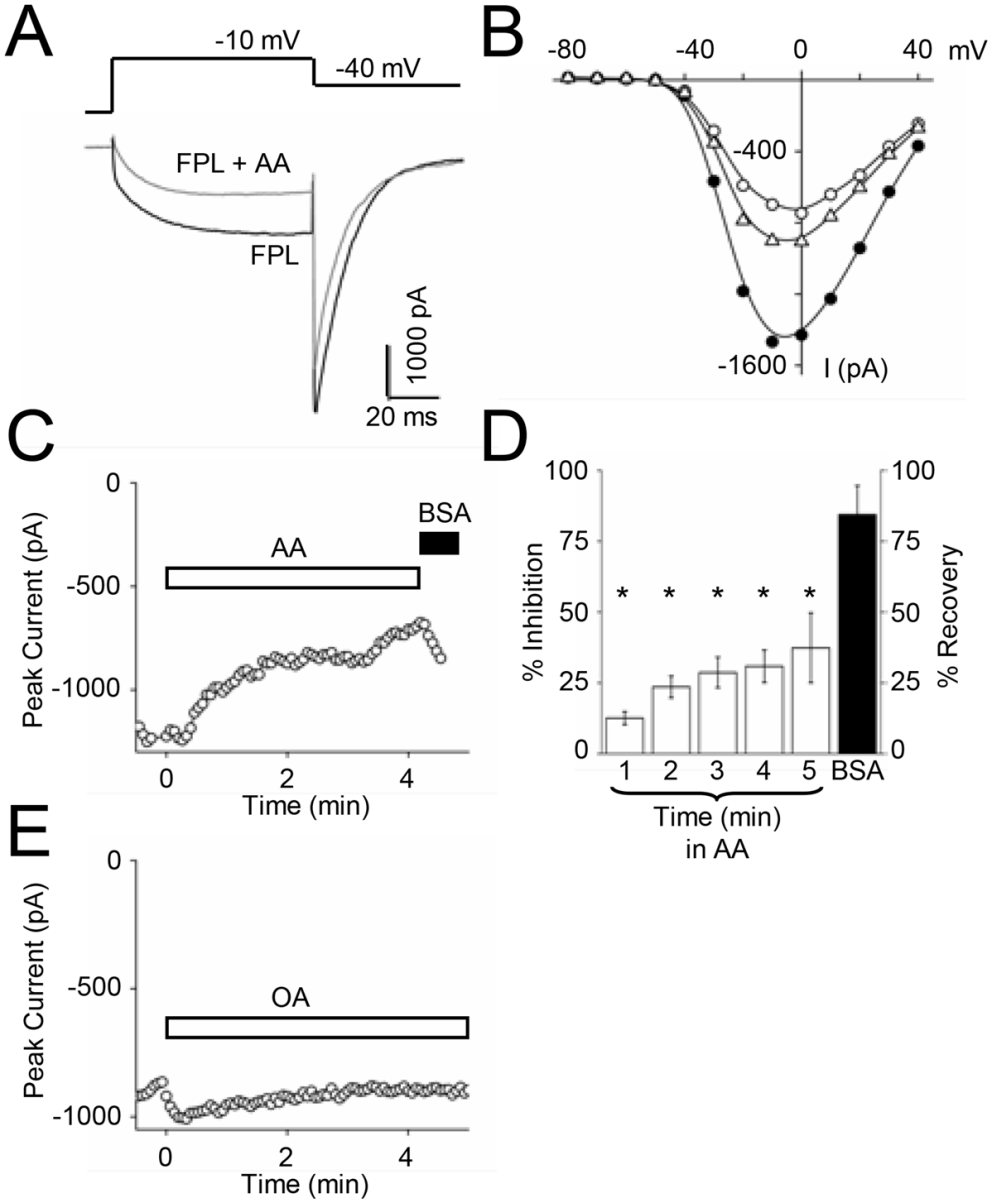
Transfected Ca_V_1.3 currents in ST14A cells are inhibited by AA. (**A**) Individual sweeps with 1 μM FPL (*black trace*), and 1 min after 10 μM AA (*dotted trace*) in the continued presence of FPL. AA inhibited both peak and tail currents; n = 4. (B) I-V relationship of recombinant current in the presence of FPL (●), FPL + AA (○), and after washing off AA with bath solution containing FPL (Δ). (C) Time course of Ca_V_1.3 current at -10 mV with 10 μM AA. After 4 min, AA was washed off with 1 mg/ml BSA. (D) Summary of Ca_V_1.3 current inhibition by AA over time (open bars) and its recovery following BSA (Black bar); *p < 0.001; n = 5–7. (E) Time course of peak Ca_V_1.3 current with 10 μM oleic acid (OA); n = 3.

To circumvent the absence of a key signaling molecule, and determine whether Ca_V_1.3 could be modulated, we directly applied exogenous AA (10 μM) to the bath and measured Ca_V_1.3 recombinant current over several minutes. In the presence of FPL, AA inhibited Ca_V_1.3 peak and long-lasting tail currents by 40 ± 12% and 29 ± 25% respectively after 1 min (n = 4). Fig 6A shows representative sweeps before (FPL) and after AA application (FPL + AA). The large variability in tail current inhibition by AA suggested that voltage may be important for this modulation. However, AA inhibited Ca_V_1.3 current at all voltages when tested over a range of test potentials as shown in the I-V plot in Fig 6B. Bovine serum albumin (BSA), which binds free fatty acids [43], reversed inhibition at all test potentials. To show that inhibition was not due to AA competing with FPL, we measured Ca_V_1.3 current in the absence of FPL prior to and after application of AA (Fig 6C) and still observed inhibition that could be reversed after adding BSA. Inhibition of Ca_V_1.3 over time by AA (open bars) and recovery by BSA (solid bar) is summarized in Fig 6D. Conversely, oleic acid (10 μM) enhanced current by 7.9 ± 0.7% after 1 minute (Fig 6E; *p* < 0.001; n = 3), suggesting that inhibition by AA is not simply the result of a nonspecific fatty acid effect. Moreover, inhibition of Ca_V_1.3 by AA demonstrates that the transfected Ca_V_1.3 LTCs in ST14A cells are capable of modulation.

## Discussion

MSNs have a resting membrane potential that oscillates between ~ -85 mV (during the “down” state) and ~-60 mV (during the “up” state) [4, 6]. Inhibition of Ca_V_1.3 is of particular interest in MSNs because it activates at potentials approximately 25 mV more negative than Ca_V_1.2, [30]. Since Ca_V_1.3 activates at the low voltage of -60 mV, this channel may open during the “up” state and contribute to reaching threshold for firing an action potential [4]. Moreover, increased D_2_R signaling or inhibition of LTCs in MSNs decreases membrane excitability [10]. MSN activity produces the only output from the striatum and thus the finely tuned regulation of MSN activity is critical for normal motor function. Disruption of MSN regulation results in severe malfunction of the basal ganglia, as seen when MSNs lose dopaminergic input in Parkinson’s disease, or when MSNs undergo cell death as seen in Huntington’s disease [5, 44].

Because studying Ca^2+^ currents in MSNs has been challenging, we searched for a cell line that might serve as a model system for MSNs. However, remarkably few neuronal cell lines have been developed despite ongoing demand for their use in biophysical studies of ion channels and in high through put systems for therapeutic drug testing. We hypothesized that ST14A cells, compared to HEK 293 cells, would express postsynaptic endogenous D_2_R signaling microdomains closely matching MSNs, thus making ST14A cells useful for studying Ca_V_1.3 modulation by D_2_R specific signaling. Therefore we examined ST14A cells to determine whether this cell line exhibits sufficient striatal properties to serve as a suitable model system for studying MSN functioning. We first tested whether ST14A cells express endogenous voltage-gated Ca^2+^ current.

We characterized a small, endogenous Ca^2+^ current from differentiated ST14A cells that develop neuronal-like processes when grown at 37°C. However, we found that only 5/15 cells had a current amplitude larger than 200 pA even in the presence of the LTC agonist FPL. This small current and the relatively low number of cells expressing a measurable current, is similar to previous findings [29]. K^+^, Na^+^ and HCN-mediated currents, in addition to the ability to fire action potentials, are also reported in a small percentage of ST14A cells [29, 45, 46]. However, when present, the Ca^2+^ current was within the lower range of current amplitudes recorded in MSNs, and was similar to L-current in MSNs in that the tail current was sensitive to LTC agonists, increasing ~ 6-fold following exposure to FPL. L-current dominates few types of neurons; one of them being MSNs (e.g., [10] [47]). Though the Ca^2+^ current in differentiated ST14A cells is small, L-current appears to make up much of the whole-cell Ca^2+^ current. Further optimization of culture conditions and/or recording conditions may increase Ca_V_1.3 expression and consequently L-current amplitudes.

As an alternative strategy to examining native Ca_V_1.3 activity in ST14A cells, we attempted to transiently transfect round ST14A cells, which lack endogenous Ca^2+^ currents, with LTC channel subunits and GFP. We didn’t know whether ST14A cells would tolerate transfection of the multiple Ca^2+^ channel subunits as well as express functional channels. However ST14A cells were transfected successfully with channel subunits and exhibited robust voltage-gated Ca^2+^ currents. We characterized the biophysical properties of isolated, recombinant Ca_V_1.3 current in a striatal-like background without the complications of primary MSNs, i.e., requiring several pharmacological blockers to silence other ionic or multiple types of Ca^2+^ currents. These large currents appeared ideal for testing whether ST14A cells would support Ca_V_1.3 current inhibition by the D_2_R agonist.

From the biochemical characterization of ST14A cells as MSN-like due to the expression of dopamine receptors (D_1_, D_2_, D_4_, D_5_) and GAD65/67 mRNAs as well as expression of D_2_R, M_1_R, G_q_α, cPLA_2_ and PP2B protein we anticipated observing LTC modulation. However, no inhibition of Ca_V_1.3 current was observed with Quin or Oxo-M. Since PLCβ-1 expression is so widespread, we tested for its expression in ST14A cells only after finding no LTC modulation by Quin. Lack of PLCβ-1 protein expression was unexpected since several intact signaling cascades are described in the growing literature regarding ST14A cells. Most notably, ST14A cells have functional CREB phosphorylation following D_2_R stimulation and Ras/MAPK, adenylyl cyclase, Wnt and JAK/STAT signaling pathways [29, 48–51]. Moreover these cells express other enzymes that act on lipids including N-acyl-phosphatidylethanolamine-hydrolyzing phospholipase D (NAPE-PLD), fatty acid amide hydrolase, diacylglyceride lipase, monoacylglycerol lipase (Bari et al., 2013), and cPLA_2_ (Fig 3C).

The absence of PLCβ-1 expression in ST14A cells coincides with the lack of Ca_V_1.3 current inhibition by either Quin or Oxo-M, supporting the importance of PLCβ-1 for both D_2_R and M_1_R signal transduction pathways [10, 52, 53]. Despite PLCβ-1’s absence, it was not obvious that no other variant of PLCβ would substitute for the missing PLCβ-1. The absence of PLCβ-1 and its apparent requirement for D_2_R signaling will be of interest to researchers who study MSNs. Interestingly, PLCβ-1 has been implicated in regulating growth and proliferation, where its absence results in uncontrolled cell proliferation [54, 55]. Thus a loss of PLCβ-1 may have occurred during the immortalization of ST14A cells.

In these experiments, we used a short splice variant of Ca_V_1.3, Ca_V_1.3b [30]. Ca_V_1.3a, which has a longer C-terminus, was not inhibited by activation of D_2_Rs when expressed in HEK 293 cells [56]; however, Ca_V_1.3a has been shown to bind the scaffolding protein Shank found in the postsynaptic density of synapses [57]. This association is necessary for Ca_V_1.3 current inhibition by D_2_Rs in primary MSNs [11]. Although the lack of the long C-terminus also could explain the absence of channel modulation by D_2_Rs in ST14A cells, Ca_V_1.3b LTCs can be modulated by both IGF-1 and AA. Exposure of SH-SY5Y cells expressing either Ca_V_1.3a or Ca_V_1.3b to IGF-1 enhances both currents and requires phosphorylation of S1486, a residue shared by both splice variants [58]. We have measured significant Ca_V_1.3b inhibition by AA in transiently transfected HEK 293 cells, consistent with a potential transmembrane site of action [27].

Since AA release occurs in the striatum from both neurons and astrocytes [59–61] following stimulation of D_2_Rs [21] or M_1_Rs [62], we tested whether bath application of AA modulated LTC in ST14A cells to be certain that Ca_V_1.3b could be modulated by molecules downstream of PLCβ-1. We were unsure whether AA would inhibit Ca_V_1.3b channels in ST14A cells similarly to Ca_V_1.3 channels in HEK 293 cells [27] or native LTCs in SCG neurons [23–25] since a wide range of actions have been reported for AA modulation of a variety of Ca^2+^ currents by other groups (see review by Roberts-Crowley et al [63]). We found that direct application of AA circumvented the lack of receptor-activated channel modulation in ST14A cells and inhibited Ca_V_1.3b recombinant current. This finding demonstrates that Ca_V_1.3 channels are capable of being modulated in ST14A cells as we have observed previously in HEK 293 cells [27]. The properties of Ca_V_1.3 inhibition by AA in HEK 293 and ST14A cells appear similar. Additionally we have found that Oxo-M inhibits currents from Ca_V_1.3b in HEK 293 cells stably transfected with M_1_Rs (unpublished data). Lastly, we have found that activation of D_2_Rs by 10 μM Quin inhibits Ca_V_2.2 currents in HEK 293 cells (unpublished data) demonstrating that this agonist protocol should be sufficient to activate D_2_Rs in ST14A cells. Therefore, the lack of current modulation by D_2_Rs or M_1_Rs, reported here, is supported by the absence of PLCβ-1 rather than an inability of Ca_V_1.3b to respond to modulation.

## Conclusions

ST14A cells are used as a model system for both the study and treatment of Huntington’s disease [64–69]. Despite the lack of D_2_R- or M_1_R-mediated Ca^2+^ channel modulation in ST14A cells reported here, this cell line was useful for elucidating that AA inhibits Ca_V_1.3 channels. We hypothesize that the consistency of AA’s inhibitory actions on Ca_V_1.3 across cell types will be of interest to researchers who study lipid signaling molecules. Moreover, we anticipate the D_2_R signaling cascade could be rescued by transfecting ST14A cells with PLCβ-1. Whether D_2_R signaling would then cause a lipid mediated inhibition of Ca_V_1.3b LTCs awaits future studies. Thus, ST14A cells are a valuable tool for studying the biophysical properties of an isolated Ca^2+^ current and the modulation of these channels by signaling molecules within the context of a striatal background to aid in understanding neuronal malfunctions of the striatum.

## References

[1] Ahlijanian MK, Westenbroek RE, Catterall WA. Subunit structure and localization of dihydropyridine-sensitive calcium channels in mammalian brain, spinal cord, and retina. Neuron. 1990;4(6):819–32. . 2163262 10.1016/0896-6273(90)90135-3

[2] Hirota K, Lambert DG. A comparative study of L-type voltage sensitive Ca^2+^ channels in rat brain regions and cultured neuronal cells. Neurosci Lett. 1997;223(3):169–72. . 9080459 10.1016/s0304-3940(97)13434-6

[3] Catterall WA, Perez-Reyes E, Snutch TP, Striessnig J. International Union of Pharmacology. XLVIII. Nomenclature and structure-function relationships of voltage-gated calcium channels. Pharmacol Rev. 2005;57(4):411–25. . 16382099 10.1124/pr.57.4.5

[4] Nicola SM, Surmeier J, Malenka RC. Dopaminergic modulation of neuronal excitability in the striatum and nucleus accumbens. Annu Rev Neurosci. 2000;23:185–215. . 10845063 10.1146/annurev.neuro.23.1.185

[5] Ehrlich ME. Huntington's disease and the striatal medium spiny neuron: cell-autonomous and non-cell-autonomous mechanisms of disease. Neurotherapeutics: the Journal of the American Society for Experimental NeuroTherapeutics. 2012;9(2):270–84. 10.1007/s13311-012-0112-2 22441874 PMC3337013

[6] Gerfen CR, Surmeier DJ. Modulation of striatal projection systems by dopamine. Annu Rev Neurosci. 2011;34:441–66. 10.1146/annurev-neuro-061010-113641 21469956 PMC3487690

[7] Hernandez-Lopez S, Bargas J, Surmeier DJ, Reyes A, Galarraga E. D1 receptor activation enhances evoked discharge in neostriatal medium spiny neurons by modulating an L-type Ca^2+^ conductance. J Neurosci. 1997;17(9):3334–42.: . 9096166 10.1523/JNEUROSCI.17-09-03334.1997 PMC6573659

[8] Surmeier DJ, Bargas J, Hemmings HC Jr., Nairn AC, Greengard P. Modulation of calcium currents by a D1 dopaminergic protein kinase/phosphatase cascade in rat neostriatal neurons. Neuron. 1995;14(2):385–97. . 7531987 10.1016/0896-6273(95)90294-5

[9] Missale C, Nash SR, Robinson SW, Jaber M, Caron MG. Dopamine receptors: from structure to function. Physiological Reviews. 1998;78(1):189–225. . 9457173 10.1152/physrev.1998.78.1.189

[10] Hernandez-Lopez S, Tkatch T, Perez-Garci E, Galarraga E, Bargas J, Hamm H, et al D2 dopamine receptors in striatal medium spiny neurons reduce L-type Ca^2+^ currents and excitability via a novel PLC[beta]1-IP3-calcineurin-signaling cascade. J Neurosci. 2000;20(24):8987–95. . 11124974 10.1523/JNEUROSCI.20-24-08987.2000 PMC6773013

[11] Olson PA, Tkatch T, Hernandez-Lopez S, Ulrich S, Ilijic E, Mugnaini E, et al G-protein-coupled receptor modulation of striatal Ca_V_1.3 L-type Ca^2+^ channels is dependent on a Shank-binding domain. J Neurosci. 2005;25(5):1050–62. . 15689540 10.1523/JNEUROSCI.3327-04.2005 PMC6725968

[12] Mink JW. Neurobiology of basal ganglia circuits in Tourette syndrome: faulty inhibition of unwanted motor patterns? Adv Neurol. 2001;85:113–22. . 11530421

[13] Levant B. Novel drug interactions at D(_2_) dopamine receptors: modulation of [^3^H]quinpirole binding by monoamine oxidase inhibitors. Life Sciences. 2002;71(23):2691–700. . 12383877 10.1016/s0024-3205(02)02109-4

[14] Ilijic E, Guzman JN, Surmeier DJ. The L-type channel antagonist isradipine is neuroprotective in a mouse model of Parkinson's disease. Neurobiology of Disease. 2011;43(2):364–71. 10.1016/j.nbd.2011.04.007 21515375 PMC3235730

[15] Kang S, Cooper G, Dunne SF, Dusel B, Luan CH, Surmeier DJ, et al Ca_V_1.3-selective L-type calcium channel antagonists as potential new therapeutics for Parkinson's disease. Nature Communications. 2012;3:1146 10.1038/ncomms2149 . 23093183

[16] Day M, Wang Z, Ding J, An X, Ingham CA, Shering AF, et al Selective elimination of glutamatergic synapses on striatopallidal neurons in Parkinson disease models. Nature Neuroscience. 2006;9(2):251–9. . 16415865 10.1038/nn1632

[17] Basselin M, Chang L, Chen M, Bell JM, Rapoport SI. Chronic carbamazepine administration attenuates dopamine D_2_-like receptor-initiated signaling via arachidonic acid in rat brain. Neurochemical Research. 2008;33(7):1373–83. 10.1007/s11064-008-9595-y . 18302021 PMC5240792

[18] Bhattacharjee AK, Meister LM, Chang L, Bazinet RP, White L, Rapoport SI. In vivo imaging of disturbed pre- and post-synaptic dopaminergic signaling via arachidonic acid in a rat model of Parkinson's disease. NeuroImage. 2007;37(4):1112–21. 10.1016/j.neuroimage.2007.06.012 17681816 PMC2040339

[19] Bhattacharjee AK, Chang L, White L, Bazinet RP, Rapoport SI. D-Amphetamine stimulates D_2_ dopamine receptor-mediated brain signaling involving arachidonic acid in unanesthetized rats. Journal of cerebral blood flow and metabolism: official journal of the International Society of Cerebral Blood Flow and Metabolism. 2006;26(11):1378–88. 10.1038/sj.jcbfm.9600290 . 16511499

[20] Bhattacharjee AK, Chang L, Lee HJ, Bazinet RP, Seemann R, Rapoport SI. D_2_ but not D_1_ dopamine receptor stimulation augments brain signaling involving arachidonic acid in unanesthetized rats. Psychopharmacology. 2005;180(4):735–42. 10.1007/s00213-005-2208-4 . 16163535

[21] Schinelli S, Paolillo M, Corona GL. Opposing actions of D_1_- and D_2_-dopamine receptors on arachidonic acid release and cyclic AMP production in striatal neurons. J Neurochem. 1994;62(3):944–9. . 8113815 10.1046/j.1471-4159.1994.62030944.x

[22] Kanterman RY, Mahan LC, Briley EM, Monsma FJ Jr., Sibley DR, Axelrod J, et al Transfected D_2_ dopamine receptors mediate the potentiation of arachidonic acid release in Chinese hamster ovary cells. Molecular Pharmacology. 1991;39(3):364–9. . 1848657

[23] Liu L, Barrett CF, Rittenhouse AR. Arachidonic acid both inhibits and enhances whole cell calcium currents in rat sympathetic neurons. American Journal of Physiology Cell Physiology. 2001;280(5):C1293–305. . 11287343 10.1152/ajpcell.2001.280.5.C1293

[24] Liu L, Rittenhouse AR. Effects of arachidonic acid on unitary calcium currents in rat sympathetic neurons. J Physiol. 2000;525 Pt 2:391–404. 10835042 10.1111/j.1469-7793.2000.00391.x PMC2269949

[25] Liu L, Zhao R, Bai Y, Stanish LF, Evans JE, Sanderson MJ, et al M_1_ muscarinic receptors inhibit L-type Ca^2+^ current and M-current by divergent signal transduction cascades. J Neurosci. 2006;26(45):11588–98. 10.1523/JNEUROSCI.2102-06.2006 . 17093080 PMC6674797

[26] Lin Z, Harris C, Lipscombe D. The molecular identity of Ca channel 1-subunits expressed in rat sympathetic neurons. Journal of Molecular Neuroscience: MN. 1996;7(4):257–67. . 8968947 10.1007/BF02737063

[27] Roberts-Crowley ML, Rittenhouse AR. Arachidonic acid inhibition of L-type calcium (Ca_V_1.3b) channels varies with accessory Ca_V_β subunits. J Gen Physiol. 2009;133(4):387–403. 10.1085/jgp.200810047 19332620 PMC2699108

[28] Cattaneo E, Conti L. Generation and characterization of embryonic striatal conditionally immortalized ST14A cells. Journal of Neuroscience Research. 1998;53(2):223–34. . 9671979 10.1002/(SICI)1097-4547(19980715)53:2<223::AID-JNR11>3.0.CO;2-7

[29] Ehrlich ME, Conti L, Toselli M, Taglietti L, Fiorillo E, Taglietti V, et al ST14A cells have properties of a medium-size spiny neuron. Experimental Neurology. 2001;167(2):215–26. . 11161610 10.1006/exnr.2000.7551

[30] Xu W, Lipscombe D. Neuronal Ca_V_1.3_1_ L-type channels activate at relatively hyperpolarized membrane potentials and are incompletely inhibited by dihydropyridines. J Neurosci. 2001;21(16):5944–51. Epub 2001/08/07. . 11487617 10.1523/JNEUROSCI.21-16-05944.2001 PMC6763157

[31] Surmeier DJ, Song WJ, Yan Z. Coordinated expression of dopamine receptors in neostriatal medium spiny neurons. J Neurosci. 1996;16(20):6579–91. . 8815934 10.1523/JNEUROSCI.16-20-06579.1996 PMC6578920

[32] Kuppers E, Sabolek M, Anders U, Pilgrim C, Beyer C. Developmental regulation of glutamic acid decarboxylase mRNA expression and splicing in the rat striatum by dopamine. Brain Research Molecular Brain Research. 2000;81(1–2):19–28. . 11000475 10.1016/s0169-328x(00)00156-x

[33] Torres JM, Gomez-Capilla JA, Ruiz E, Ortega E. Semiquantitative RT-PCR method coupled to capillary electrophoresis to study 5alpha-reductase mRNA isozymes in rat ventral prostate in different androgen status. Mol Cell Biochem. 2003;250(1–2):125–30. . 12962150 10.1023/a:1024902419502

[34] Liu L, Roberts ML, Rittenhouse AR. Phospholipid metabolism is required for M_1_ muscarinic inhibition of N-type calcium current in sympathetic neurons. European Biophysics Journal: EBJ. 2004;33(3):255–64. 10.1007/s00249-003-0387-7 . 15004729

[35] Yue DT, Backx PH, Imredy JP. Calcium-sensitive inactivation in the gating of single calcium channels. Science. 1990;250(4988):1735–8. . 2176745 10.1126/science.2176745

[36] Chien AJ, Carr KM, Shirokov RE, Rios E, Hosey MM. Identification of palmitoylation sites within the L-type calcium channel β2a subunit and effects on channel function. The Journal of Biological Chemistry. 1996;271(43):26465–8. . 8900112 10.1074/jbc.271.43.26465

[37] Yan Z, Flores-Hernandez J, Surmeier DJ. Coordinated expression of muscarinic receptor messenger RNAs in striatal medium spiny neurons. Neuroscience. 2001;103(4):1017–24. . 11301208 10.1016/s0306-4522(01)00039-2

[38] Moreland RB, Nakane M, Donnelly-Roberts DL, Miller LN, Chang R, Uchic ME, et al Comparative pharmacology of human dopamine D_2_-like receptor stable cell lines coupled to calcium flux through G_qo5_ . Biochem Pharmacol. 2004;68(4):761–72. . 15276084 10.1016/j.bcp.2004.05.019

[39] Bitner RS, Nikkel AL, Otte S, Martino B, Barlow EH, Bhatia P, et al Dopamine D_4_ receptor signaling in the rat paraventricular hypothalamic nucleus: Evidence of natural coupling involving immediate early gene induction and mitogen activated protein kinase phosphorylation. Neuropharmacology. 2006;50(5):521–31. . 16324724 10.1016/j.neuropharm.2005.10.009

[40] Ahlgren-Beckendorf JA, Levant B. Signaling mechanisms of the D_3_ dopamine receptor. J Recept Signal Transduct Res. 2004;24(3):117–30. . 15521358 10.1081/rrs-200029953

[41] Fishburn CS, Elazar Z, Fuchs S. Differential glycosylation and intracellular trafficking for the long and short isoforms of the D_2_ dopamine receptor. The Journal of Biological Chemistry. 1995;270(50):29819–24. . 8530376 10.1074/jbc.270.50.29819

[42] Lin JY, Chung KK, de Castro D, Funk GD, Lipski J. Effects of muscarinic acetylcholine receptor activation on membrane currents and intracellular messengers in medium spiny neurones of the rat striatum. Eur J Neurosci. 2004;20(5):1219–30. . 15341594 10.1111/j.1460-9568.2004.03576.x

[43] Spector AA. Fatty acid binding to plasma albumin. Journal of Lipid Research. 1975;16(3):165–79. . 236351

[44] Herrero MT, Barcia C, Navarro JM. Functional anatomy of thalamus and basal ganglia. Childs Nerv Syst. 2002;18(8):386–404. . 12192499 10.1007/s00381-002-0604-1

[45] Strauss U, Bajorat R, Mueller J, Rolfs A, editors. "Hidden" Sodium Currents in Neuronal Progenitor Cells. 2003: Washington, DC: Society for Neuroscience, Online.

[46] Bajorat R, Brauer AU, Wasner U, Rolfs A, Strauss U. Functional significance of HCN2/3-mediated I(h) in striatal cells at early developmental stages. Journal of Neuroscience Research. 2005;82(2):206–13. . 16175581 10.1002/jnr.20643

[47] Martella G, Madeo G, Schirinzi T, Tassone A, Sciamanna G, Spadoni F, et al Altered profile and D_2_-dopamine receptor modulation of high voltage-activated calcium current in striatal medium spiny neurons from animal models of Parkinson's disease. Neuroscience. 2011;177:240–51. 10.1016/j.neuroscience.2010.12.057 . 21195752

[48] Varani K, Rigamonti D, Sipione S, Camurri A, Borea PA, Cattabeni F, et al Aberrant amplification of A_2A_ receptor signaling in striatal cells expressing mutant huntingtin. FASEB journal: official publication of the Federation of American Societies for Experimental Biology. 2001;15(7):1245–7. . 11344102

[49] Bottcher T, Mix E, Koczan D, Bauer P, Pahnke J, Peters S, et al Gene expression profiling of ciliary neurotrophic factor-overexpressing rat striatal progenitor cells (ST14A) indicates improved stress response during the early stage of differentiation. Journal of Neuroscience Research. 2003;73(1):42–53. . 12815707 10.1002/jnr.10624

[50] Cattaneo E, De Fraja C, Conti L, Reinach B, Bolis L, Govoni S, et al Activation of the JAK/STAT pathway leads to proliferation of ST14A central nervous system progenitor cells. The Journal of Biological Chemistry. 1996;271(38):23374–9. . 8798541 10.1074/jbc.271.38.23374

[51] Lange C, Mix E, Rateitschak K, Rolfs A. Wnt signal pathways and neural stem cell differentiation. Neurodegener Dis. 2006;3(1–2):76–86. . 16909041 10.1159/000092097

[52] Wu L, Bauer CS, Zhen XG, Xie C, Yang J. Dual regulation of voltage-gated calcium channels by PtdIns(4,5)P_2_ . Nature. 2002;419(6910):947–52. . 12410316 10.1038/nature01118

[53] Liu L, Rittenhouse AR. Arachidonic acid mediates muscarinic inhibition and enhancement of N-type Ca^2+^ current in sympathetic neurons. Proceedings of the National Academy of Sciences of the United States of America. 2003;100(1):295–300. 10.1073/pnas.0136826100 12496347 PMC140955

[54] Cocco L, Follo MY, Faenza I, Billi AM, Ramazzotti G, Martelli AM, et al Inositide signaling in the nucleus: from physiology to pathology. Advances in Enzyme Regulation. 2010;50(1):2–11. Epub 2009/11/10. 10.1016/j.advenzreg.2009.10.007 . 19895834

[55] Cocco L, Follo MY, Faenza I, Fiume R, Ramazzotti G, Weber G, et al Physiology and pathology of nuclear phospholipase C beta1. Advances in Enzyme Regulation. 2011;51(1):2–12. Epub 2010/11/03. 10.1016/j.advenzreg.2010.09.015 . 21035488

[56] Bell DC, Butcher AJ, Berrow NS, Page KM, Brust PF, Nesterova A, et al Biophysical properties, pharmacology, and modulation of human, neuronal L-type (_1D_, Ca_V_1.3) voltage-dependent calcium currents. Journal of Neurophysiology. 2001;85(2):816–27. . 11160515 10.1152/jn.2001.85.2.816

[57] Zhang H, Maximov A, Fu Y, Xu F, Tang TS, Tkatch T, et al Association of Ca_V_1.3 L-type calcium channels with Shank. J Neurosci. 2005;25(5):1037–49. 10.1523/JNEUROSCI.4554-04.2005 . 15689539 PMC6725973

[58] Gao L, Blair LA, Salinas GD, Needleman LA, Marshall J. Insulin-like growth factor-1 modulation of CaV1.3 calcium channels depends on Ca^2+^ release from IP_3_-sensitive stores and calcium/calmodulin kinase II phosphorylation of the 1 subunit EF hand. J Neurosci. 2006;26(23):6259–68. Epub 2006/06/10. 10.1523/jneurosci.0481-06.2006 . 16763033 PMC6675183

[59] Zanassi P, Paolillo M, Schinelli S. Coexpression of phospholipase A_2_ isoforms in rat striatal astrocytes. Neurosci Lett. 1998;247(2–3):83–6. . 9655598 10.1016/s0304-3940(98)00262-6

[60] Rouach N, Tence M, Glowinski J, Giaume C. Costimulation of N-methyl-D-aspartate and muscarinic neuronal receptors modulates gap junctional communication in striatal astrocytes. Proceedings of the National Academy of Sciences of the United States of America. 2002;99(2):1023–8. . 11792837 10.1073/pnas.022257499 PMC117424

[61] Blanchet F, Gauchy C, Perez S, Glowinski J, Kemel ML. Role of arachidonic acid in the regulation of the NMDA-evoked release of acetylcholine in striatal compartments. Synapse. 1999;31(2):140–50. . 10024011 10.1002/(SICI)1098-2396(199902)31:2<140::AID-SYN7>3.0.CO;2-2

[62] Tence M, Cordier J, Premont J, Glowinski J. Muscarinic cholinergic agonists stimulate arachidonic acid release from mouse striatal neurons in primary culture. The Journal of Pharmacology and Experimental Therapeutics. 1994;269(2):646–53. . 8182531

[63] Roberts-Crowley ML, Mitra-Ganguli T, Liu L, Rittenhouse AR. Regulation of voltage-gated Ca^2+^ channels by lipids. Cell Calcium. 2009;45(6):589–601. 10.1016/j.ceca.2009.03.015 19419761 PMC2964877

[64] Weinelt S, Peters S, Bauer P, Mix E, Haas SJ, Dittmann A, et al Ciliary neurotrophic factor overexpression in neural progenitor cells (ST14A) increases proliferation, metabolic activity, and resistance to stress during differentiation. Journal of Neuroscience Research. 2003;71(2):228–36. . 12503085 10.1002/jnr.10477

[65] Rigamonti D, Bauer JH, De-Fraja C, Conti L, Sipione S, Sciorati C, et al Wild-type huntingtin protects from apoptosis upstream of caspase-3. J Neurosci. 2000;20(10):3705–13. . 10804212 10.1523/JNEUROSCI.20-10-03705.2000 PMC6772672

[66] Zuccato C, Tartari M, Crotti A, Goffredo D, Valenza M, Conti L, et al Huntingtin interacts with REST/NRSF to modulate the transcription of NRSE-controlled neuronal genes. Nat Genet. 2003;35(1):76–83. . 12881722 10.1038/ng1219

[67] Bosch M, Pineda JR, Sunol C, Petriz J, Cattaneo E, Alberch J, et al Induction of GABAergic phenotype in a neural stem cell line for transplantation in an excitotoxic model of Huntington's disease. Experimental Neurology. 2004;190(1):42–58.: . 15473979 10.1016/j.expneurol.2004.06.027

[68] Bari M, Battista N, Valenza M, Mastrangelo N, Malaponti M, Catanzaro G, et al In vitro and in vivo models of Huntington's disease show alterations in the endocannabinoid system. The FEBS Journal. 2013;280(14):3376–88. 10.1111/febs.12329 . 23659592

[69] Ermak G, Hench KJ, Chang KT, Sachdev S, Davies KJ. Regulator of calcineurin (RCAN1-1L) is deficient in Huntington disease and protective against mutant huntingtin toxicity in vitro. The Journal of Biological Chemistry. 2009;284(18):11845–53.: 10.1074/jbc.M900639200 19270310 PMC2673253

